# The Role of Extracellular Vesicles and PIBF in Embryo-Maternal Immune-Interactions

**DOI:** 10.3389/fimmu.2018.02890

**Published:** 2018-12-13

**Authors:** Julia Szekeres-Bartho, Sandra Šućurović, Biserka Mulac-Jeričević

**Affiliations:** ^1^Department of Medical Biology and Central Electron Microscope Laboratory, Medical School, Pécs University, Pécs, Hungary; ^2^János Szentágothai Research Centre, Pécs University, Pécs, Hungary; ^3^Endocrine Studies, Centre of Excellence, Pécs University, Pécs, Hungary; ^4^MTA-PTE Human Reproduction Research Group, Pécs, Hungary; ^5^Department of Physiology and Immunology, Faculty of Medicine, University of Rijeka, Rijeka, Croatia

**Keywords:** pregnancy, progesterone, PIBF, NK cells, cytokines, extracellular vesicles

## Abstract

Pregnancy represents a unique immunological situation. Though paternal antigens expressed by the conceptus are recognized by the immune system of the mother, the immune response does not harm the fetus. Progesterone and a progesterone induced protein; PIBF are important players in re-adjusting the functioning of the maternal immune system during pregnancy. PIBF expressed by peripheral pregnancy lymphocytes, and other cell types, participates in the feto-maternal communication, partly, by mediating the immunological actions of progesterone. Several splice variants of PIBF were identified with different physiological activity. The full length 90 kD PIBF protein plays a role in cell cycle regulation, while shorter splice variants are secreted and act as cytokines. Aberrant production of PIBF isoforms lead to the loss of immune-regulatory functions, resulting in and pregnancy failure. By up regulating Th2 type cytokine production and by down-regulating NK activity, PIBF contributes to the altered attitude of the maternal immune system. Normal pregnancy is characterized by a Th2-dominant cytokine balance, which is partly due to the action of the smaller PIBF isoforms. These bind to a novel form of the IL-4 receptor, and induce increased production of IL-3, IL-4, and IL-10. The communication between the conceptus and the mother is established via extracellular vesicles (EVs). Pre-implantation embryos produce EVs both *in vitro*, and *in vivo*. PIBF transported by the EVs from the embryo to maternal lymphocytes induces increased IL-10 production by the latter, this way contributing to the Th2 dominant immune responses described during pregnancy.

## Introduction

Fifty per cent of the antigens expressed by the fetus originate from the father. Therefore, they are recognized as foreign and should be “rejected,” yet in spite of all odds, the maternal immune system does not attack the fetus.

The immune system of the mother must comply with two conflicting requirements, i.e., while creating a favorable environment for the developing fetus, it has to be prepared to control possible emerging infections. By establishing a delicate balance, the foeto-maternal unit is able to satisfy the interests of both the mother and the fetus. Progesterone, and its mediator the progesterone-induced blocking factor (PIBF) are important players in this process. In addition to its endocrine effects, progesterone also acts as an “immunosteroid” (1). Progesterone induces Th2 differentiation of established T cell clones (2) and regulates the homing and activity of uterine NK cells (3), among others, by upregulating HLA-G gene expression (4), which is the ligand for both NK inhibitory and activating receptors. Many of the immunological effects of progesterone are mediated by PIBF.

This review aims to give an overview on the diverse roles of progesterone and PIBF in re-setting the functions of the maternal immune system, and on extracellular vesicles (EVs) as means of establishing the communication between the two sides of the feto-maternal unit.

## Progesterone Receptors

The biological activity of progesterone is mediated by genomic and non-genomic pathways. The former depends on two nuclear progesterone receptor (PR) isoforms, PRA, and PRB (5, 6). Both isoforms are the products of the same gene, but their transcription is controlled by two distinct promoters (7).

Mice lacking PRA are infertile (8, 9), while the PRB isoform mediates the effects of progesterone on mammary gland development (10). The reproductive tissue responses to progesterone depend on the relative expression of the two isoforms (11). Progesterone can also signal through membrane-bound PRs or via the MAPK or PI3K/Akt pathway. The latter entirely bypasses the classical PR pathway, signaling either through the JNK pathway or by increasing cAMP (12).

**Figure 1:**
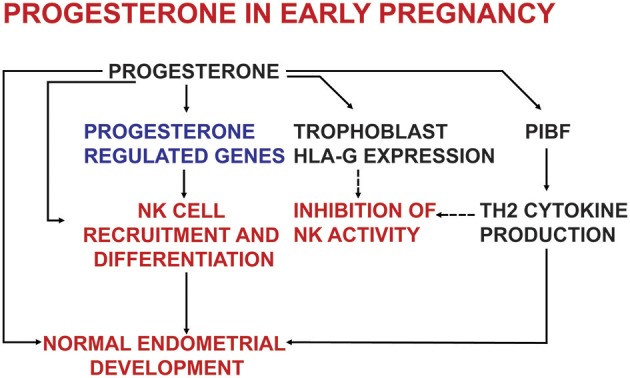
The effects of progesterone on endometrial development and on the immune system in early pregnancy.

Studies on PR knock out mice revealed, that PRs are required not only for endometrial receptivity and decidualization (13), but also for establishing an appropriate immune environment in the endometrium (14) (Figure 1). Several studies using nuclear and cytosol binding assays and immunohistochemistry—indicate, that in certain conditions lymphoid cells might express PRs (15–20).

Peripheral lymphocytes of pregnant women, but not those of non-pregnant individuals express PRs (21, 22). Earlier we demonstrated an inverse relationship between progesterone binding capacity and cytotoxic activity of peripheral human lymphocytes (23). The cytotoxic activity of pregnancy lymphocytes was significantly reduced by progesterone at concentrations comparable to those, present in pregnancy serum, while 100-fold higher progesterone concentrations were required to alter the cytotoxic activity of lymphocytes from non-pregnant individuals (24). These findings already suggested that pregnancy lymphocytes might contain progesterone binding sites, which enable them to respond to progesterone.

The number of PR positive cells increases throughout normal gestation. In women with recurrent miscarriage, or in those, showing clinical symptoms of threatened pre-term delivery, the % of PR expressing cells among peripheral lymphocytes, is significantly lower than in women with uneventful pregnancies (21, 22). These findings suggest, that the presence of PR positive lymphocytes is required for a normally progressing pregnancy.

PR expression in peripheral lymphocytes or lymphoid cell lines has been confirmed by several studies (15–17, 25, 26). Both classical PR isoforms are present in peripheral blood NK cells (18), however, PR expression in decidual NK cells is controversial. Van den Heuvel et al. (3) demonstrated PRs in murine decidual NK cells, while Henderson et al. (27) failed to detect of PRs in purified decidual NK cells. Nevertheless, the majority of decidual NK cells are PIBF positive (28).

Both *in vitro* and *in vivo* activation of human non-pregnancy lymphocytes result in increased PR expression (29, 30). Paternal leukocyte immunization of women with recurrent miscarriage also increases the number of PR expressing lymphocytes (31).

**Figure 2:**
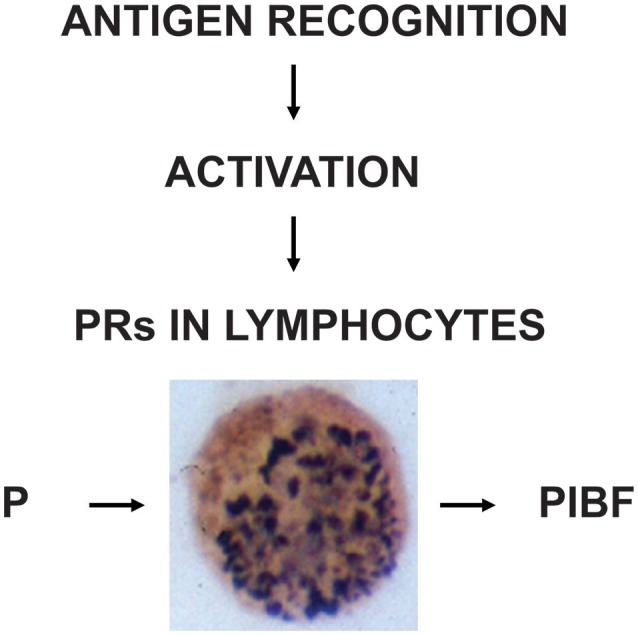
The induction and biological significance of lymphocyte progesterone receptors. Following recognition of fetal antigens, maternal lymphocytes become activated and express progesterone receptors (PR). The presence of PRs enables the cells, to respond to progesterone (P), e.g., by PIBF production.

These data indicate that PR expression is a characteristic feature of activated immune cells (Figure 2).

## Progesterone-Regulated Genes

Among the progesterone-regulated genes, the transcription factors Hox-A10, Hox-A11, and the glican binding protein galectin-1 (Gal-1) are the most relevant for the feto- maternal immunological interaction (32). Hox-A10 deficient mice are characterized by a polyclonal T cell proliferation (33), and impaired decidual NK cell differentiation (24, 25, 34, 35).

Gal-1 expression in the female reproductive system was described in the nineties, and recently, many functional aspects of this lectin during pregnancy have been discovered (36–38). Gal-1 gene expression in the mouse uterine tissues has been shown to be regulated by ovarian steroids during implantation (39). In line with this, Than et al. (40) identified an estrogen response element in the Gal-1 gene.

Altered Gal-1 expression in the placenta has been implicated in several pregnancy pathologies.

Proteomic studies showed that Gal-1 expression is reduced in placental villous tissues from patients with spontaneous miscarriages (41). On the other hand, placental Gal-1 expression was found to be increased in severe preeclampsia (42) as well as in chorioamnionitis (43), possibly representing a fetal response to an exaggerated systemic maternal inflammation.

In pregnant mice, stress-induced Gal-1-deficiency results in an increased rate of fetal loss, which is corrected by progesterone exposure. Gal-1 treatment on the other hand, prevents the stress-induced decrease of progesterone as well as PIBF levels, and restores the resorption rates to a normal level (44). These data suggest a cross-regulation between progesterone and Gal-1 at the foeto-maternal interface.

PIBF is another progesterone-regulated gene. The mouse PIBF1 gene, is transcribed to 16 different mRNAs, the longest of which is 3,677 bp long and includes 18 exons. The predicted protein is a 90 kDa molecule, composed of 756 amino acids (45). The full-length PIBF protein shows a peri-nuclear localization, (46) and has been identified as a component of the peri-centriolar satellite (47), suggesting its role in cell cycle regulation. Alternative splicing produces several smaller isoforms, which are localized in the cytoplasm (45) and are accountable for the immunological effects of PIBF.

In murine pregnancy, embryo resorption as well as term delivery are associated with the absence or lower expression of the N terminal PIBF exons, which might have important functional consequences (48).

The loss of the N-terminal exons results in a significantly reduced production of the full length protein, and also prevents the synthesis of the smaller protein isoforms, which act on the cytokine pattern and NK activity (45).

## The Immuno-Modulating Effects of PIBF and the Maintenance of Pregnancy

PIBF was first described as a 34 kDa protein produced by activated pregnancy lymphocytes (30). It has become evident since, that PIBF might be expressed by various reproductive tissues as well as malignant tumors (49–51). A human study illustrated that trophoblast cells in the placenta could express PIBF proteins of 30, 50, and 90 kDa in first trimester (52).

Several human studies suggest an association between PIBF levels and the outcome of pregnancy. In a prospective cohort study attempting to identify early risk factors for miscarriage, PIBF was one of the factors showing a strong association with miscarriage risk (53). In normal human pregnancy, both serum-and urinary PIBF concentrations increase during gestation, while in women, with miscarriage, or preterm labor, urinary PIBF levels fail to increase (54). Preterm birth was predictable by lower than normal pregnancy PIBF values mesaured at 24–28gestational week (55), but not at 11–13 weeks of gestation (56), suggesting, that predictive value of PIBF determination depends on the interval, between sampling and the onset of labor. In line with this, progestogen-treatment of women with threatened miscarriage corrected the initially low PIBF levels, and in parallel, reduced the miscarriage rate to a similar level of healthy controls (57).

While the full length PIBF has been shown to regulate trophoblast and tumor cell invasiveness (58–60), the smaller isoforms are secreted, bind to the PIBF receptor (39, 61) and via their cytokine-like functions, play a role in the materno-fetal relationship, both in animal models and in humans.

Some of the immunological effects of progesterone, e.g., that on NK activity and cytokine balance, are mediated by PIBF.

Earlier studies showed that in mice PIBF protects pregnancy by controlling NK activity (62). Anti-PIBF treatment of pregnant mice results in increased resorption, which are corrected by simultaneously neutralizing NK activity with anti-NK antibodies (62).

Decidual NK cells, are functionally different from their circulating counterparts. Though decidual NK cells selectively overexpress perforin and granzymes A and B (41, 63), their cytotoxic activity is low. In normal pregnancy decidual NK cells contribute to creating a favorable environment for placentation, implantation and embryo development (64), yet they are equipped with cytotoxic molecules, to fight intrauterine infections (65, 66).

In the day 12 mouse decidua, there is an abundance of PIBF positive granulated cells. These cells are missing from the deciduae of alymphoid mice, but when alymphoid mice are reconstituted of with bone marrow from male BALB/c mice, the PIBF positive granulated cells re-appear in the decidua. These data suggest that the PIBF+ cells belong to the lymphoid lineage, and based on their DBA lectin reactivity, to the group of NK cells.

PIBF+ NK cells contain perforin, which co-localizes with PIBF in the cytoplasmic granules. In day 12.5 normal mouse pregnancy only 54% of the PIBF + decidual NK cells contain perforin, whereas in PIBF deficient mice of the same gestational age, not only do most of the PIBF + NK cells disappear, but all of the remaining ones are perforin positive (28).

This implies that in mice PIBF exerts a pregnancy protective effect by keeping NK activity under restraint.

The local mechanism of the protective action of PIBF is less easily studied in humans, than in animal models. Nevertheless, a recent study showed that the otherwise scarcely studied decidual B cells produce PIBF under the effect of IL-33, and that these PIBF + B cells are missing from the choriodecidual area of women with pre-term labor (67) (Nature).

In spite of their high perforin content, spontaneous cytotoxic activity of human decidual NK cells is moderate (68). Progesterone inhibits human NK cytolytic activity *in vitro* (19), and upregulates HLA-G gene expression (4). Because HLA-G is a ligand for NK inhibitory and activating receptors, upregulation of HLA-G by progesterone might be one of the pathways accounting for the low cytotoxic activity of decidual NK cells.

Decidual NK activity appears to be affected by PIBF. PIBF inhibits upregulation of perforin expression in activated human decidual NK cells and prevents degranulation (69, 70).

Though there is no evidence that NK cells directly attack the trophoblast, recurrent miscarriage is often accompanied by increased decidual NK activity (71–75), suggesting that this mechanism might be a factor in the underlying pathology of repeated pregnancy loss.

It is well-established, that while normally progressing pregnancies are characterized by a Th2 dominant cytokine pattern, an excess of Th1-associated cytokines leads to pregnancy termination (76, 77). In humans, recurrent miscarriages are associated with a Thl-dominant peripheral cytokine profile (78–82).

Both progesterone and PIBF play a role in the induction of the Th2 biased cytokine balance. In the presence of progesterone resting human peripheral blood T cells differentiate into Th2-like clones, furthermore, progesterone treatment of Th1-like T cell clones shifts the cytokine production of these cells toward Th2 (2). Neutralization of endogenous PIBF activity in pregnant mice by specific anti-PIBF antibody terminates pregnancy, reduces the synthesis of IL-10, and increases that of IFN-γ (83).

**Figure 3:**
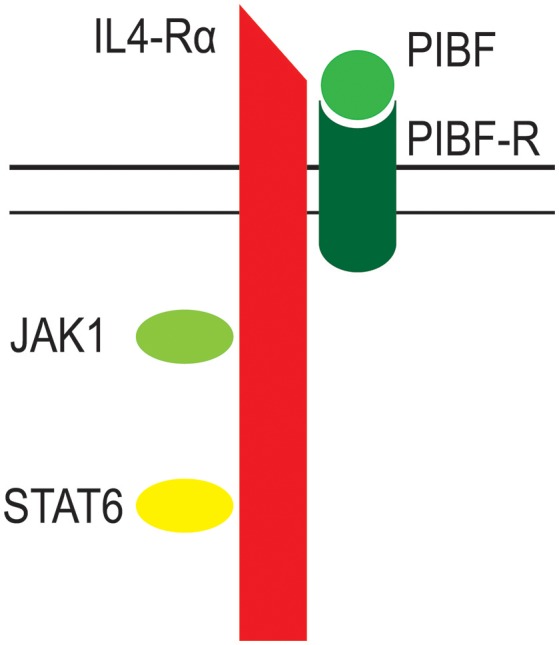
The structure of the PIBF receptor. The receptor for PIBF is a GPI anchored protein. After PIBF binding the receptor associates with the alpha chain of the IL-4 receptor. This initiates intracellular signaling, via the Jak/STAT pathway.

The PIBF receptor is a glycosylphosphatidylinositol (GPI)-anchored protein, which, for signaling, temporarily associates with the alpha chain of the IL-4 receptor (39, 61). Engagement of the PIBF receptor results in immediate STAT6 activation, whereas, a 24 h incubation with progesterone is needed to phosphorylate STAT6, indicating, that the effect of progesterone on Th2 cytokine production is mediated by PIBF (61) (Figure 3).

By signaling via this novel form of the IL-4 receptor (39, 61), PIBF induces increased production of IL-3, IL-4, and IL-10 by activated murine lymphocytes (84).

Raghupathy et al. (78, 79) investigated the production of Th1 and Th2 cytokines by progesterone treated peripheral blood lymphocytes isolated from women with recurrent miscarriage. They showed that progestogen induced PIBF production down-regulates the production of Thl-type cytokines and stimulates the production of Th2-type cytokines. Furthermore, progestogen treatment of women with pre-term delivery induces a Th2 dominant cytokine pattern (78, 79).

Taken together, these data suggest, that by up regulating Th2 type cytokine production and by down-regulating NK activity PIBF affects the immune response in a way, which might have an impact on the foeto-maternal relationship.

## The Peri-Implantation Embryo Communicates With the Maternal Immune System Via Extracellular Vesicles

Earlier studies described a communication between the embryo and the maternal immune system. Embryo culture media were shown to exert an immunosuppressive activity (84). In line with this, incubation of human peripheral lymphocytes with the culture media of fertilized eggs, but not with follicular fluid resulted in increased IL-10 mRNA expression by the lymphocytes (85).

These data suggest that embryo derived signals, can influence the maternal immune response, however, the mechanism of signal transport has not been thoroughly investigated.

In recent years EVs have received much attention. These membrane-coated structures may express phosphatidylserine (PS) in their membrane (86), which reacts with Annexin V. EVs are categorized by their origin and size (87). Exosomes are 30–100 nm, and originate from internalized endocytic vesicles. Microvesicles (100 nm^−1^ μm in diameter), are shed from the plasma membrane by budding, and apoptotic vesicles (1–5 μm in diameter) are released from cells undergoing apoptosis (88).

All types of cells produce EVs which transport various cargos, (including proteins, nucleic acids, and lipids) from one cell to the other. Proteins, e.g., cytokines carried and released by EVs could initiate signaling pathways, and thus alter the biological functions of the target cells (89, 90).

EVs might be considered as candidates for conveying the information from the embryo to the mother. The message carried by EVs has been shown to affect the reproductive process at different points.

EVs have been demonstrated in mouse oocytes (91) as well as in the follicular fluid (92–96) and extra villous trophoblast (97). The tetraspanins CD9 and CD81 expressed by oocyte derived EVs have been suggested to play a role in sperm-oocyte membrane fusion (98–100). Follicular fluid exosomes contain miRNAs, some of them targeting genes that regulate oocyte growth (95) as well as different pathways of reproduction, and endocrine functions (94).

EV—mediated interactions between the endometrium and the blastocyst promote implantation (101). In sheep endometrium, EV production is controlled by progesterone, and endometrium derived EVs were shown to reach the embryo, (102).

EVs from a human uterine epithelial cells express the extracellular matrix metalloprotease inducer (103) which induces the expression of MMPs, thus EVs might also play a role in endometrial remodeling (101, 103, 104).

EVs can be produced by virtually all cell types, however it has been debated, whether a single embryo would be able to produce a detectable amount of EVs. The more so, because the culture medium contains serum or serum albumin, both of which could also be a source of EVs. In a review Tannetta et al. (105) points out the difficulty of measuring EVs in embryo culture medium.

Now there is evidence, that pre-implantation embryos produce EVs both *in vitro* and *in vivo* (106).

Earlier we showed that spent media of *in vitro* cultured human embryos contain a significantly higher number of EVs, than empty media, and the number of nucleic acid containing EVs in day 5 human embryo culture media, might serve as an indicator of embryo competence (106). Other groups have also reported the presence of EVs in embryo culture medium. It is now obvious that embryos release EVs, which are taken up by close by cells (90). Giacomini et al. (107) characterized HLA-G containing EVs isolated from conditioned media from *in vitro* cultured human embryos. EVs were demonstrated in the culture medium of bovine blastocyst and the characteristics of these EVs varied depending on embryo competence (108). Qu et al. (109) showed that the negative effects of culture media replacement during embryo culture are due to the loss of embryo derived EVs, and can be corrected by exosome supplementation. This suggests, that embryo derived EVs do indeed carry molecules that promote normal embryo development.

Embryo-derived EVs might also communicate with the maternal immune system by presenting antigens (110, 111), carrying MHC molecules (112–115), or cytokines (116–121). HLA-G-positive EVs isolated from the plasma from healthy term pregnant women have been reported to bind to T lymphocytes (122), and moderately decrease peripheral T lymphocyte STAT3 phosphorylation (122). EVs at the same time can induce pro-inflammatory cytokines and chemokines in primary macrophage cultures (123, 124).

EVs bind to CD8+ and–though to a lesser degree to CD4+ lymphocytes-, via the phosphatidylserine—phosphatidylserine receptor interaction (125). CD4+ and CD8+ cells express similar numbers of phosphatidylserine receptors, therefore, it is likely, that in addition to the phosphatidylserine—phosphatidylserine receptor interaction, other, yet unidentified mechanisms might also be involved in binding of EVs to CD8+ cells. With immuno- electron microscopy we identified PIBF in embryo-derived EVs, and showed that these PIBF containing EVs might affect the immune response (125).

Incubation of murine spleen cells with embryo-derived EVs, increased the number of IL-10+ cells among peripheral CD8+ cells, but not in the CD4+ population. IL-10 producing CD8+ T lymphocytes might moderate antigen-induced inflammatory responses, since these cells have been shown to control influenza virus induced inflammation in the foet (126), and to prevent liver damage during chronic hepatitis C virus infection (127).

Pre-treatment of EVs with an anti-PIBF antibody abrogates the above described effect of the EVs. These data suggest that PIBF transported by the EVs from the embryo to maternal lymphocytes might induce increased IL-10 production by the latter, this way contributing to the Th2 dominant immune responses described during pregnancy. The finding is in line with our earlier data, (83) showing increased IL-10 production of murine spleen cells in the presence of PIBF.

**Figure 4:**
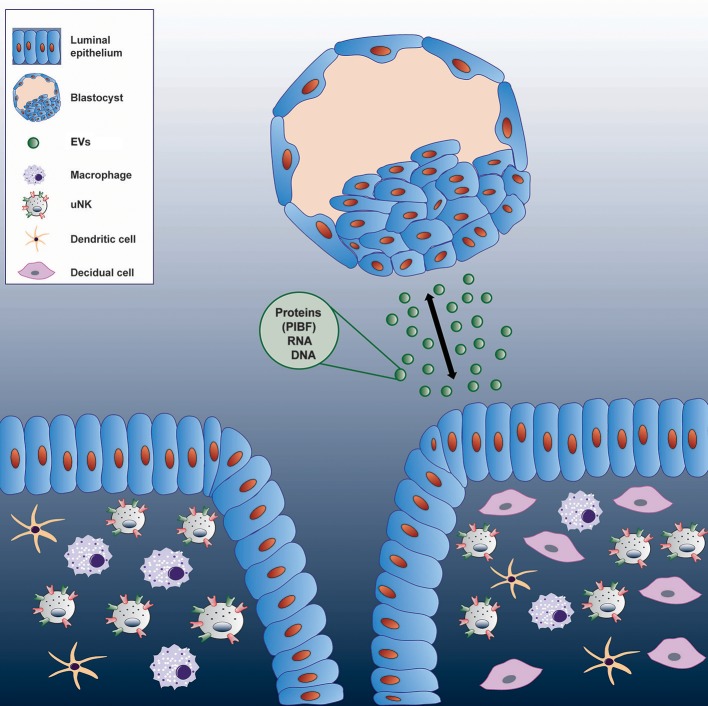
Communication between the embryo and the maternal immune system via extracellular vesicles.

This pathway might have its significance in reproduction. Because embryo derived EVs transport various molecules, - PIBF, among others-, it cannot be ruled out, that these structures act as means of feto-maternal or materno-fetal communication in the peri-implantation period (Figure 4).

## Author Contributions

JS-B wrote the paper. BM-J, SŠ, and JS-B designed and performed the experiments.

### Conflict of Interest Statement

The authors declare that the research was conducted in the absence of any commercial or financial relationships that could be construed as a potential conflict of interest. The handling editor is currently co-organizing a Research Topic with one of the authors JS-B and confirms the absence of any other collaboration.
